# Methylation vs. Protein Inflammatory Biomarkers and Their Associations With Cardiovascular Function

**DOI:** 10.3389/fimmu.2020.01577

**Published:** 2020-07-31

**Authors:** Héléne Toinét Cronjé, Hannah R. Elliott, Cornelie Nienaber-Rousseau, Fiona R. Green, Aletta E. Schutte, Marlien Pieters

**Affiliations:** ^1^Centre of Excellence for Nutrition, North-West University, Potchefstroom, South Africa; ^2^MRC Integrative Epidemiology Unit, University of Bristol, Bristol, United Kingdom; ^3^Population Health Sciences, Bristol Medical School, University of Bristol, Bristol, United Kingdom; ^4^Faculty of Health and Medical Sciences, Formerly School of Biosciences and Medicine, University of Surrey, Guildford, United Kingdom; ^5^Hypertension in Africa Research Team, Medical Research Council Unit for Hypertension and Cardiovascular Disease, North-West University, Potchefstroom, South Africa; ^6^School of Public Health and Community Medicine, University of New South Wales, George Institute for Global Health, Sydney, NSW, Australia

**Keywords:** cell counts, epigenetics, epidemiology, inflammation, neutrophil-to-lymphocyte, lymphocyte-to-monocyte

## Abstract

DNA methylation data can be used to estimate proportions of leukocyte subsets retrospectively, when directly measured cell counts are unavailable. The methylation-derived neutrophil-to-lymphocyte and lymphocyte-to-monocyte ratios (mdNLRs and mdLMRs) have proven to be particularly useful as indicators of systemic inflammation. As with directly measured NLRs and LMRs, these methylation-derived ratios have been used as prognostic markers for cancer, although little is known about them in relation to other disorders with inflammatory components, such as cardiovascular disease (CVD). Recently, methylation of five genomic cytosine-phosphate-guanine sites (CpGs) was suggested as proxies for mdNLRs, potentially providing a cost-effective alternative when whole-genome methylation data are not available. This study compares seven methylation-derived inflammatory markers (mdNLR, mdLMR, and individual CpG sites) with five conventionally used protein-based inflammatory markers (C-reactive protein, interleukins 6 and 10, tumor-necrosis factor alpha, and interferon-gamma) and a protein-based inflammation score, in their associations with cardiovascular function (CVF) and risk. We found that markers of CVF were more strongly associated with methylation-derived than protein-based markers. In addition, the protein-based and methylation-derived inflammatory markers complemented rather than proxied one another in their contribution to the variance in CVF. There were no strong correlations between the methylation and protein markers either. Therefore, the methylation markers could offer unique information on the inflammatory process and are not just surrogate markers for inflammatory proteins. Although the five CpGs mirrored the mdNLR well in their capacity as proxies, they contributed to CVF above and beyond the mdNLR, suggesting possible added functional relevance. We conclude that methylation-derived indicators of inflammation enable individuals with increased CVD risk to be identified without measurement of protein-based inflammatory markers. In addition, the five CpGs investigated here could be useful surrogates for the NLR when the cost of array data cannot be met. Used in tandem, methylation-derived and protein-based inflammatory markers explain more variance than protein-based inflammatory markers alone.

## Introduction

Methylation-derived cell count ratios, particularly methylation-derived neutrophil-to-lymphocyte and lymphocyte-to-monocyte ratios (mdNLRs and mdLMRs), are increasingly being used as robust alternatives to flow cytometry-based cell count ratios as indicators of systemic inflammation (1–3). One key advantage is that they can be derived from archived blood in cohorts where cytometric measurements have not been performed (1, 4). Through unique methylation signatures, leukocyte subtypes can be separated and quantified with comparative accuracy. Validation analyses have reported an *R*^2^ estimate of at least 0.95 when methylation-derived estimates of leukocyte sub-types and NLRs are regressed on those measured directly (1, 5).

Similar to cytometry-based ratios, mdNLRs and mdLMRs are considered prognostic markers of overt inflammatory diseases such as rheumatoid arthritis (3) and cancer (2, 6). However, little is known about these methylation-derived ratios in relation to less pronounced inflammatory diseases such as cardiovascular disease (CVD). While directly measured cell counts have been established as indicators of CVD severity, recurrence, and prognosis (7–12), the use of cell counts, methylation-derived or directly measured, in CVD risk prediction and disease progression in the epidemiological setting is less well-known. There is also no consensus on the reference ranges or thresholds to be used when characterizing the NLR or MLR as healthy, at risk, or pathological (13), with large ethnic diversity also being reported (14–16). More recently, methylation levels of five cytosine-phosphate-guanine sites (CpGs), namely cg25938803, cg10456459, cg01591037, cg03621504, and cg00901982, have been suggested as proxy markers for the mdNLR, because of their robust associations with myeloid cell (neutrophil and monocyte) differentiation (2, 4). If true, measurement of this small panel of CpGs could render whole genome methylation measurement unnecessary in cohorts with limited financial resources.

Blood-based protein inflammatory markers, such as C-reactive protein (CRP), interleukin-6 (IL-6), and tumor necrosis factor alpha (TNF-α), are useful epidemiological tools for quantifying inflammatory state and disease risk (17). However, cell count ratios are considered superior to circulating inflammatory markers in their ability to quantify systemic inflammation (7, 18, 19). Cell count ratios provide a more integrated view of systemic inflammation by reflecting the relative contribution of the innate (neutrophils/monocytes as indicators of general inflammation) and adaptive (lymphocytes as an indicator of physiological stress) immune responses (19), supporting their use in population-based research. Because few cohorts have access to data on both cell counts and protein-based inflammatory markers, we set out to determine whether cell count ratios provide added benefit in characterizing inflammatory status and CVD risk independent from protein-based inflammatory markers in our cohort where both are measured. The rapid advancement of epigenetic investigations in CVD research, together with the increasing number of samples with epigenetic data available, including increasing ethnic diversity among available samples (20, 21), also motivate our interest in exploring novel ways to mine for valuable additional information from previously analyzed samples.

To this end, we investigated methylation-derived and protein-based biomarkers of inflammation in relation to cardiovascular function (CVF) in a cohort of black South African men. We included seven methylation-derived (mdNLR, mdLMR, and the five myeloid CpGs) and five high-sensitivity protein-based [CRP, IL-6, IL-10, TNF-α, interferon [IFN]-γ] markers of inflammation. In addition, we used a protein-based inflammation score (22) to provide the combined effect of inflammatory biomarkers. First, we compared how well the protein-based and methylation-derived inflammatory markers reflected CVD risk according to literature-based cut-offs. We also compared the mdNLRs and mdLMRs reported in this sample population to ratios reported in studies on healthy individuals from different ethnicities. This is followed by an investigation of the relationship between the methylation-derived and protein-based inflammatory biomarkers in our study population, and a comparison of their relative associations with CVF markers. Lastly, we investigated whether a combination of methylation and protein inflammatory biomarkers provided added benefit in explaining CVF variance, or whether one proxies the other. Cardiovascular function is represented by blood pressure (BP), heart rate (HR), and arterial stiffness. In contrast to previous work, we evaluated the methylation-derived biomarkers in a population-based cohort as opposed to a case-control design, to yield better understanding of the value these markers may have in the general population.

## Methods

### Study Population

This is a cross-sectional investigation of 120 self-identified Batswana men who were enrolled in the North West province, South African arm of the international Prospective Urban and Rural Epidemiology study (PURE-SA-NW) in 2015 (23). Individuals were randomly selected from 926 participants residing in selected rural and urban regions, based on the following criteria: male sex, testing negative for the human-immunodeficiency virus at the time of data collection, and bio-sample availability. These criteria were incorporated to minimize confounding by sex and CD4+ cell counts. The PURE-SA-NW study received ethical approval from the Health Research Ethics Committee of the North-West University, South Africa (NWU-00016–10-A1). Written informed consent was obtained from participants prior to data collection.

### DNA Methylation, Cell Counts, and Cell Count Ratios

Genomic DNA isolated from peripheral whole blood was bisulfite-converted prior to genome-wide methylation quantification using the Illumina Infinium MethylationEPIC BeadChip according to the manufacturer's protocol (Illumina®, San Diego, CA, USA). Details regarding DNA extraction, quality control, methylation quantification, data processing, and data normalization have been reported previously (24). Sample cell fractions were estimated using the IDOL optimized L-DMR library for whole blood samples in the *FlowSorted.Blood.EPIC* R software package (5). Neutrophil counts were divided by lymphocytes (calculated as the sum of B-, CD4T, CD8T, and natural killer cell counts), and lymphocytes by monocytes, to obtain the respective mdNLRs and mdLMRs (1, 2).

### Inflammatory Markers

Fasting blood samples were collected in ethylenediamine tetra acetic acid tubes for the analysis of cytokines and in anti-coagulant-free tubes for the quantification of CRP. Samples were centrifuged within 30 min of collection at 2,000 × g for 15 min. The Cobas^©^ Integra 400 (Roche^©^ Clinical System, Roche Diagnostics, Indianapolis, IN) was used to quantify high-sensitivity CRP concentrations. High-sensitivity Q-Plex™ planar-based multiplexed enzyme-linked immunosorbent assays (Quansys Biosciences, Logan, UT) were performed to measure IL-6, IL-10, TNF-α, and IFN-γ. An inflammation summary score was calculated to amalgamate related inflammatory proteins as suggested previously (22, 25). Data on CRP, IL-6, IL-10, TNF-α, and IFN-γ were log_e_-transformed to improve distribution. Thereafter data were converted to z-scores to account for the difference in measurement units. The average of the z-scores is reported here as the inflammatory score.

### Measures of Cardiovascular Function

Systolic and diastolic BP (SBP and DBP) and HR were measured using the OMRON M6 device (Omron Healthcare, Kyoto, Japan). Participants were seated in an upright position with legs uncrossed. After participants had rested for 10 min, the correct cuff size was fitted on their right arms, whereafter two measurements were recorded with a 5-min interval. Data from the second measurement were used for analysis. Pulse pressure (PP) was then calculated as the difference between SBP and DBP. Large artery stiffness was investigated using the current gold standard measurement, carotid-femoral pulse wave velocity [cfPWV (26)], using the SphygmoCor XCEL device (AtCorMedical Pty. Ltd., Sydney, Australia). The transit-distance method was used to measure PWV along the descending thoracoabdominal aorta. Two readings were taken from each participant while supine. Data from the second reading were used.

### Cardiovascular Risk Factors (Co-variates)

Socio-demographic information and data on medicine use and smoking habits, alcohol consumption, and physical activity were collected by interview, using a standardized and validated questionnaire (23). Current smoking and alcohol consumption status were reported as *current, former*, or *never*, but has been dichotomized here to *never* and *ever* (where *ever* denotes *formerly* and *currently*). Participants also reported the frequency and quantity of usage, age at the start of use, and previous attempts at abstinence. Participants were asked by interviewers to provide information on any prescribed or over-the-counter medication they regularly make use of. Body mass index (BMI) was calculated as weight (measured using an electronic scale) per unit height (measured using a stadiometer) squared (kg/m^2^). Waist circumference was measured using a steel tape (Lufkin, Cooper Tools, Apex NC, USA), according to standard anthropometric procedures. Physical activity is reported as a continuous physical activity index measure determined using data from a modified Baecke's questionnaire validated for use in South African adults (27).

Blood samples for the subsequent analyses were collected and processed in the same manner as described above. Fasting glucose, total cholesterol, triglycerides, and low- and high-density lipoprotein cholesterol (LDL-C and HDL-C) were quantified with the Cobas^©^ Integra 400 (Roche^©^ Clinical System, Roche Diagnostics, Indianapolis, IN). The Cobas^©^ Integra 400 plus (Roche^©^, Basel, Switzerland) was used to determine serum gamma-glutamyl transferase concentrations. Glycated hemoglobin was quantified using the D-10 Hemoglobin testing system from Bio-Rad^©^ (#220-0101).

### Statistical Analysis

Analyses were performed using version 3.5.0 of R statistical software (28). Data distribution was evaluated using Shapiro-Wilks tests and visual inspection of histograms and quantile-quantile plots. As most of the data were not normally distributed, we proceeded with non-parametric testing where appropriate. Prior to linear modeling, all skewed data were log_e_-transformed. A Bonferroni adjustment, based on the number of independent comparisons, was used to account for multiple testing. Variables were considered dependent when the coefficient of determination (*R*^2^) between them was >0.2. Based on these criteria, we regarded the number of independent inflammatory markers tested here as four ([1] methylation-derived markers, [2] CRP, [3] IL-6 and IL-10, and [4] TNF-α and IFN-γ) and the CVF markers as three [[1] BP markers, [2] HR, and [3] cfPWV].

**Figure 1:**
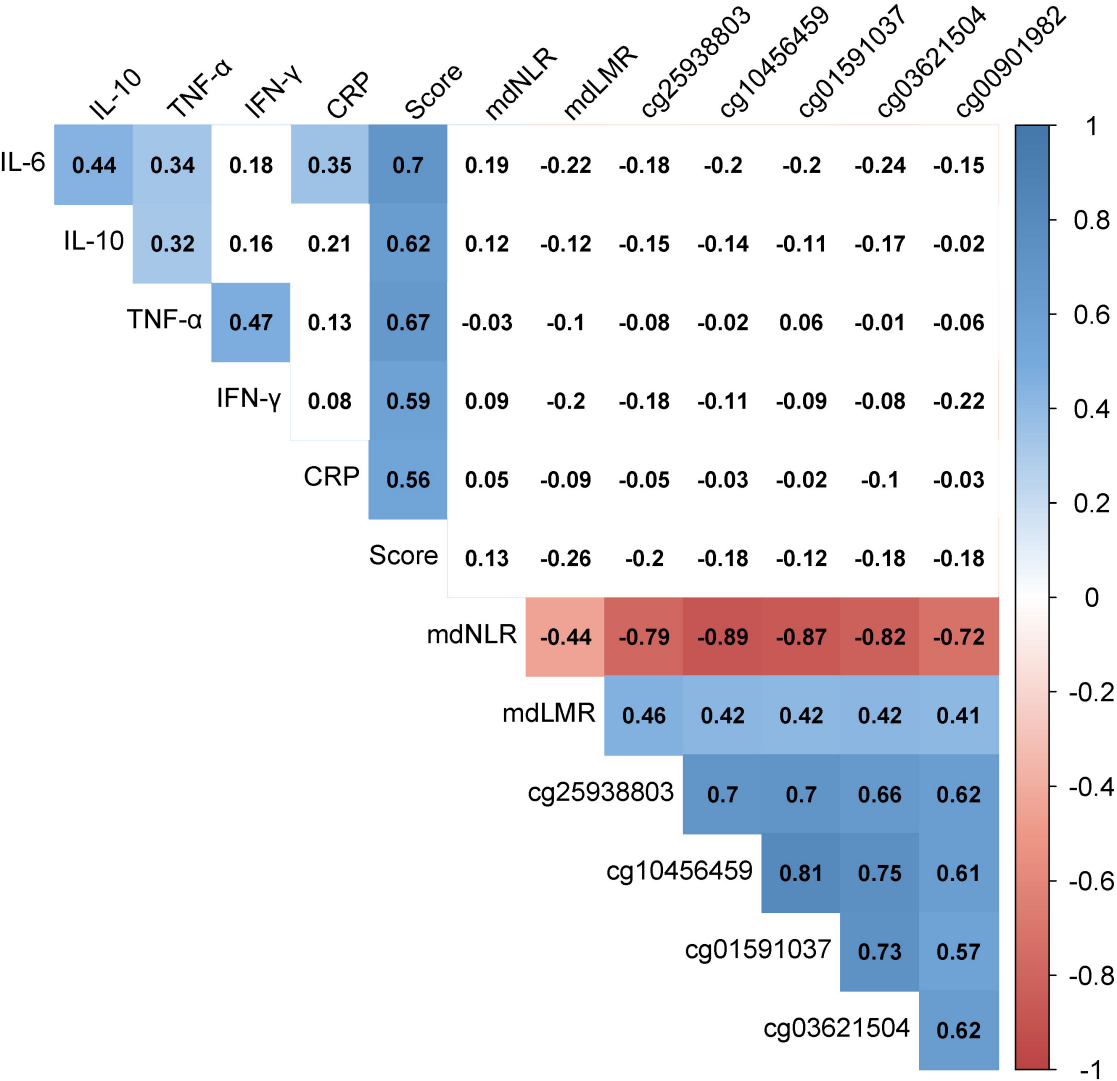
Heat map of the partial Spearman correlations among protein-based and methylation-derived biomarkers of inflammation. Numeric values indicate Spearman's rho values while controlling for age and smoking status. The presence of color indicates *p* < 0.003 (α = 0.05/16, calculated as 4 × 4 independent inflammatory marker comparisons). The shades of color represent the strength and direction of the correlation. “Score” represents the average of the IL-6, IL-10, TNA-α, IFN-γ, and CRP z-scores. CRP, C-reactive protein; IFN-γ, interferon-gamma; IL-6, interleukin-6; IL-10, interleukin-10; mdLMR, methylation-derived lymphocyte-to-monocyte ratio; mdNLR, methylation-derived neutrophil-to-lymphocyte ratio; TNF-α, tumor necrosis factor-alpha.

Relationships among the biomarkers of inflammation were assessed with partial Spearman correlations, controlling for age and smoking status (*Hmisc* R package for quantification and *corrplot* for visualization, Figure 1), because of the well-described association of age and smoking status with methylation (29, 30). The Bonferroni threshold for these correlations was *p* < 0.003 (α = 0.05/16 tests, calculated as 4 × 4 independent inflammatory marker comparisons).

Next, the associations between individual inflammatory biomarkers (protein-based and methylation-derived) and CVF, and the variance in CVF explained by these inflammatory markers, were investigated using linear multivariate regression models adjusted for known cardiovascular risk markers (**Table 2** and Supplementary Table 1). The Bonferroni threshold for these models was set at *p* < 0.004 (α = 0.05/12 tests, calculated as 4 independent inflammatory × 3 independent CVF markers).

Thereafter, a combination of methylation-derived inflammatory markers (selected using backwards-stepwise regression models) were investigated in similar linear multivariate regression models (using the *car* and *relaimpo* packages), this time adjusting for known cardiovascular risk markers including inflammation, represented by the protein-based inflammatory score (**Table 3** and Supplementary Table 3). The Bonferroni threshold was 0.02 (0.05/3, calculated as three independent CVF markers × 1 independent inflammatory marker). The relative contribution of inflammatory biomarkers to the CVF variance was determined using the *relaimpo* package's *lmg* metric from the *calc.relimp* function (31). Chi-square tests were used to compare linear models, before and after adding methylation-derived biomarkers (**Table 3**).

To identify covariates, known cardiovascular risk markers were entered in backward stepwise linear regression models with CVF markers as outcome, to identify risk markers strongly associated with CVF in this study population: age, dwelling place (rural/urban), body composition (BMI and waist circumference), level of education, physical activity, smoking status, self-reported alcohol consumption status, and medicine use. In addition, blood lipid levels (total cholesterol, LDL-C, HDL-C, and triglycerides), markers of glucose metabolism (fasting glucose and glycated hemoglobin), and gamma-glutamyl transferase were tested. With the exception of dwelling place, smoking and alcohol consumption status, and medicine use, all variables were investigated as continuous variables. Only risk markers retained by the stepwise regression models were adjusted for in subsequent models to avoid over fitting, given our limited sample size. Based on these results, we made use of two main covariate clusters in all regression analyses. First (hereafter referred to as Model 1), we adjusted for age only. Second (hereafter referred to as Model 2), we adjusted for age, smoking status, dwelling place (rural/urban), BMI, LDL-C, HDL-C, and medicine use (as a binary variable, yes or no). When cfPWV was the outcome, mean arterial pressure was additionally adjusted for in both models. In **Table 3**, inflammation, quantified using the inflammatory score, was added to Model 2 and is referred to as Model 3.

## Results

The clinical characteristics of our cohort are provided in Table 1 and Supplementary Table 1. We report on 120 ostensibly healthy men, aged between 45 and 88 years (x = 63). Sixty-nine of these men resided in rural areas, 79 reported regular medication use, and 64 classified themselves as *ever* smokers.

**Table 1:** Descriptive characteristics of the study population according to their CVD risk.

**Clinical characteristics**	**Median (25%; 75%)**	**Increased CVD risk cut-off**	**References**	Individuals at increased risk N* (%)
**PROTEIN-BASED INFLAMMATORY MARKERS**				
CRP (mg/L)	3.00 (1.52; 7.90)	>3.0	(17)	60/119 (50.4)
IFN-γ (pg/mL)	1.51 (0.76; 2.79)			
IL-6 (pg/mL)	3.97 (2.10; 7.47)	>1.5	(32)	104/118 (89.7)
IL-10 (pg/mL)	3.53 (2.88; 4.86)			
TNF-α (pg/mL)	10.1 (7.68; 13.1)			
**METHYLATION-DERIVED CELL RATIO MARKERS**				
MdNLR	1.34 (0.90; 1.71)	>1.8^&^	(33)	26/120 (21.7)
MdLMR	4.30 (3.39; 4.88)	<4.3^&^	(34)	60/120 (50)
cg25938803 (β)	0.32 (0.26; 0.38)			
cg10456459 (β)	0.38 (0.31; 0.47)			
cg01591037 (β)	0.38 (0.32; 0.45)			
cg03621504 (β)	0.25 (0.19; 0.34)			
cg00901982 (β)	0.30 (0.21; 0.35)			
**MARKERS OF CARDIOVASCULAR FUNCTION**				
SBP (mmHg)	137 (122; 147)	>140	(35)	50/120 (41.6)
DBP (mmHg)	83.0 (77.0; 94.0)	>90		40/120 (33.3)
PP (mmHg)	49.0 (41.8; 60.3)	≥60		36/120 (30.0)
HR (bpm)	68.0 (58.0; 82.0)	>80		31/120 (25.8)
cfPWV (m/s)	9.35 (8.30; 10.5)	>10		47/111 (42.3)
**CARDIOVASCULAR RISK MARKERS**				
BMI (kg/m^2^)	21.2 (18.7; 25.3)	>25	(36)	37/117 (31.6)
LDL-C (mmol/L)	2.47 (1.77; 3.15)	≥2.60	(37)	51/120 (42.5)
HDL-C (mmol/L)	1.29 (0.99; 1.65)	<1.00		31/120 (25.8)

* *Expressed as number of participants at increased risk out of the number of participants with data for the specific variable ^&^Directly measured cell count ratio cut-off. DBP, diastolic blood pressure; SBP, systolic blood pressure; PP, pulse pressure; CRP, C-reactive protein; cfPWV, carotid-femoral pulse wave velocity; IFN-γ, interferon-gamma; IL-6, interleukin-6; IL-10, interleukin-10; IQR, interquartile range; mdLMR, methylation-derived lymphocyte-to-monocyte ratio; mdNLR, methylation-derived neutrophil-to-lymphocyte ratio; HR, heart rate; TNF-α, tumor necrosis factor-alpha*.

Table 1 also indicates, where available, literature-based cut-off values for increased CVD risk, in the same unit as reported in our cohort. Based on the CVF and cardiovascular risk markers reported in Table 1, 25–50% of our study population was at increased CVD risk. Regarding the protein-based inflammatory markers, CRP reflected a similar risk (50%), while IL-6 cut-offs categorized almost 90% of the study population as suffering from low-grade inflammation (25, 38) and increased CVD risk. No reference ranges for IL-10, TNA-α, or IFN-γ in terms of chronic low-grade inflammation or CVD risk are established.

The methylation-derived cell ratios were in agreement with the CVD risk portrayed by CRP and the CVF and CVD risk markers (Table 1). The mdNLR and mdLMR, respectively, classified 21% and 50% of the PURE-SA-NW participants as having increased CVD risk. Nineteen participants (16%) were classified as at higher CVD risk by both ratios. Supplementary Figures 1, 2 depict the methylation-derived cell count ratios observed in the PURE-SA-NW men (blue) in relation to directly measured reference ranges published for healthy individuals from several ethnic groups (green) and cut-offs previously used to predict the odds of specific CVD outcomes, or ranges from patients in case-control studies (orange). On average, the PURE-SA-NW cohort had comparatively lower mdNLRs than the NLR ranges reported in other population-based cohorts. The PURE-SA-NW cohort also exhibited only slight overlap with the patient groups reported. In terms of the mdLMR (where a higher ratio is more favorable), comparatively lower ratios were observed than those reported in other population-based cohorts. The PURE-SA-NW mdLMR range also spanned the LMRs of the three CVD patient cohorts.

### Relationship Between Biomarkers of Inflammation

Figure 1 depicts the relationship between the 13 investigated indicators of inflammation, adjusted for age and smoking status. Positive correlations were observed among the protein biomarkers. The strongest correlations were between IL-6 and IL-10 (*r* = 0.44, *p* = 9.9 × 10^−7^) and between TNF-α and IFN-γ (*r* = 0.47, *p* = 1.1 × 10^−7^). All inflammatory proteins also associated strongly with the inflammatory score (*r* > 0.55, *p* < 1.6 × 10^−10^ for all). Comparatively stronger correlations were observed among the methylation-derived biomarkers. The negative CpG-mdNLR and positive CpG-mdLMR correlation coefficients reflect the positive associations of these CpGs with monocytes (*r* > 0.27, *p* < 0.004 in all instances) and lymphocytes (*r* > 0.70, *p* < 1.0 × 10^−18^ for all), and the negative association with neutrophils (*r* < −0.72, *p* < 2 × 10^−20^ in all instances). No convincing evidence for protein-methylation correlations was observed.

### Association of Biomarkers of Inflammation With Markers of Cardiovascular Function

Supplementary Table 2 reports the partial Spearman correlation coefficients among the protein-based and methylation-derived biomarkers of inflammation and markers of CVF, adjusted for age. No evidence was observed for a linear relationship between BP (SBP, DBP, and PP) and inflammatory markers (protein-based and methylation-derived). Comparatively stronger correlations were observed where HR and cfPWV were concerned, particularly in relation to the methylation-derived inflammatory biomarkers. The only Bonferroni significant correlations observed (*p* ≤ 0.004) were between cg25938803 and HR (*r* = −0.27, *p* = 0.003), cg25938803 and cfPWV (*r* = −0.29, *p* = 0.002), and cg10456459 and HR (*r* = −0.30, *p* = 0.001).

Summary statistics of the linear regression models quantifying the relative contribution of the investigated inflammatory biomarkers to the variance in CVF markers are shown in Table 2. Only inflammatory biomarkers that contributed to these models (either Model 1 or 2) at a Bonferroni-adjusted significance threshold of p < 0.004 are reported in Table 2. Test statistics for all 13 investigated inflammatory markers and the five CVF markers (SBP, DBP, PP, HR, and cfPWV) are reported in Supplementary Table 3.

**Table 2:** Variance in cardiovascular function explained by individual inflammatory biomarkers.

**Inflammatory biomarker**	**Model**	**CVF variance explained by covariates**	**Inflammatory biomarker's contribution to the model**		**Variance explained by full model (including the inflammatory biomarker)**
			**β (25%; 75%)**	***P***	
**HR (log bpm)**					
CRP	1	0%	0.04 (0.01; 0.07)	0.006	7%
	2	9%	0.05 (0.02; 0.07)	**0.001**	19%
mdNLR	1	0%	0.11 (0.04; 0.18)	**0.003**	7%
	2	9%	0.10 (0.02; 0.18)	0.01	15%
mdLMR	1	0%	−0.19 (−0.32; −0.07)	**0.003**	8%
	2	9%	−0.18 (−0.30; −0.07)	0.005	16%
cg10456459	1	0%	−0.20 (−0.32; −0.08)	**0.002**	8%
	2	9%	−0.17 (−0.29; −0.08)	0.008	15%
cg03621504	1	0%	−0.12 (−0.21; −0.04)	**0.004**	7%
	2	9%	−0.11 (−0.19; −0.04)	0.02	14%
**cfPWV (log m/s)**					
cg25938803	1	25%	−0.20 (−0.31; −0.09)	**3.8E−04**	33%
	2	36%	−0.18 (−0.29; −0.09)	**0.002**	42%
cg03621504	1	25%	−0.12 (−0.19; −0.04)	**0.002**	31%
	2	36%	−0.09 (−0.16; −0.04)	0.01	40%

*All inflammatory and CVF biomarkers reported were log_e_-transformed prior to analysis. Regression coefficient interpretation should be that one per cent change in x (inflammatory marker) will induce a regression coefficient (β) per cent change in y (CVF marker). Model 1: CVF marker ~ (inflammatory biomarker) + age; Model 2: CVF marker ~ (inflammatory biomarker) + age + smoking status + dwelling place + smoking status + BMI + LDL-C + HDL-C + medication use. When cfPWV was the outcome, mean arterial pressure was additionally adjusted for. p ≤ 0.004 highlighted in bold. CRP: C-reactive protein; cfPWV: carotid-femoral pulse wave velocity; mdLMR: methylation-derived lymphocyte-to-monocyte ratio; mdNLR: methylation-derived neutrophil-to-lymphocyte ratio; HR: heart rate*.

Markers of CVF appeared to be more strongly associated with methylation-derived inflammatory markers than with protein-based biomarkers (Table 3, Supplementary Table 3). Regarding relationships with HR, four methylation-derived biomarkers reached Bonferroni cut-off, although the associations were attenuated when additional covariates were added to the model. The association between HR and CRP, on the other hand, strengthened upon full adjustment (Model 2). Two CpGs, cg25938803 and cg03621504, associated negatively with cfPWV. Both associations attenuated on full adjustment with evidence remaining for the association between cg25938803 and cfPWV. To specifically determine the impact of medications impacting the cardiovascular system we repeated our analysis and replaced total medication use with CVD medication use (detailed in Supplementary Table 1). No attenuation of associations was observed upon doing so.

**Table 3:** The additive value of methylation-derived inflammatory biomarkers to known cardiovascular risk markers in relation to cardiovascular function.

Regression model*	**Inflammatory biomarker**			**Variance explained**	***X^**2**^ p-value***
	**β (25%; 75%)**	**P**	**Contribution to CVF variance^&^**		
**SBP (log mmHg)**					
Model 3				14%	**0.005**
+mdNLR	0.11 (0; 0.23)	0.05	2.2%	**22%**	
+cg03621504	0.21 (0.08; 0.35)	**0.003**	7.3%		
**cfPWV (log m/s)**					
Model 3				41%	**0.008**
+mdNLR	−0.13 (−0.26; −0.003)	0.05	1.5%	**48%**	
+ cg25938803	−0.24 (−0.43; −0.06)	**0.01**	4.7%		
+ cg03621504	−0.11 (−0.24; 0.02)	0.10	1.6%		

All inflammatory and CVF biomarkers reported were log_e_-transformed prior to analysis. Regression coefficient interpretation should be that one per cent change in x (inflammatory marker) will induce a regression coefficient (β) per cent change in y (CVF marker). *Model 3: CVF marker ~ age + smoking status + dwelling area + BMI + LDL-C + HDL-C + medicine use + score (the average of the IL-6, IL-10, TNA-α, IFN-γ and CRP z-scores). When cfPWV was the outcome, mean arterial pressure was additionally adjusted for. ^&^lmg metric providing a decomposition of the model explained variance into non-negative contributions (31). X^2^ p value = Chi-square p value when the regression models with and without methylation-derived inflammatory biomarkers are compared. SBP: systolic blood pressure; cfPWV: carotid-femoral pulse wave velocity; mdNLR: methylation-derived neutrophil-to-lymphocyte ratio. p ≤ 0.02 highlighted in bold.

### Additive Value of Methylation-Derived Inflammatory Markers When Investigating Cardiovascular Function

Next, we investigated whether the methylation-derived inflammatory markers can increase the variance explained in CVF when added to a model containing known CVD risk markers, including inflammation. To this end, we included the protein-based inflammatory score, as an amalgamated biomarker of inflammation in the covariate list of Model 2 (referred to below as Model 3). To identify which methylation-derived biomarkers to investigate, we performed a backward stepwise regression analysis for all CVF phenotypes. Age (and mean arterial pressure when cfPWV was the outcome) and the seven methylation-derived inflammatory biomarkers were added to these models as independent variables. Only the markers retained by the backward stepwise regression were included in further analyses (Table 3). For all three BP-related markers, cg03621504 and mdNLR were retained. For HR, cg25938803, cg10456459, and cg01591037 were retained. For cfPWV, cg25938803, cg03621504, and mdNLR were retained. The additive variance explained was determined when the retained methylation markers were added to a fully adjusted (Model 3) multivariate regression analysis. Table 3 reports only the model for which the addition of methylation biomarkers increased the explained variance at a Bonferroni-adjusted threshold of p < 0.02. Full summary statistics are provided in Supplementary Table 4.

Myeloid CpGs, cg03621504, and cg25938803 were the only methylation-derived markers that had strong evidence of individual contribution to the variance in SBP and cfPWV, respectively. The retained methylation-derived inflammatory markers contributed an additional ~7% to the variance explained in SBP and cfPWV, after age, smoking status, body composition, blood lipids, socio-economic status (represented by urban/rural status), medication use and inflammation (protein-based) had been accounted for. For every ~2% methylation increase in cg03621504 a 10 mmHg change in the geometric mean of SBP was observed. For cfPWV, an increase of 1 m/s in the geometric mean resulted from a ~3% methylation increase in cg25938803. Again, findings remained robust upon replacing total medication use with CVD medication use.

## Discussion

In this study we report the value of methylation-derived indicators of inflammation in relation to CVF, including BP and large artery stiffness. Although the methylation-derived and protein-based inflammatory markers did not demonstrate strong associations with each other, both reflected a similar degree of increased CVD risk. Methylation-derived markers appeared to be more strongly associated with CVF than the protein-based inflammatory markers tested. Furthermore, when exploring models explaining variance in CVF, we found that methylation biomarkers, particularly the myeloid CpGs, explained variance in addition to variance already explained by known CVD risk markers, including inflammation reflected by a protein-based inflammatory score. This suggests that methylation-derived inflammatory markers may complement protein-based inflammatory markers in explaining CVF marker variance, rather than simply being a proxy thereof.

### mdNLR and mdLMR in the PURE-SA-NW Cohort

Although the evidence for appropriate cut-off values for increased CVD rather than overt disease risk remains unclear, a meta-analysis of 38 studies, investigating NLRs in relation to stroke, acute coronary syndrome, coronary artery disease, and a composite of these events (33), provides some guidelines. According to these guidelines, 21% of the PURE-NW cohort can be classified as at increased CVD risk. This is lower than the risk percentage indicated by CRP and IL-6 concentrations, but in agreement with the other CVF and CVD risk markers investigated, albeit in the lowest range of risk prediction. When comparing our CVD risk and mdNLRs with the individual cohorts depicted in Supplementary Figure 1, some discrepancies are, however, noted. Almost 60% of the PURE-SA-NW study population can be classified as suffering from hypertension (according to BPs and anti-hypertensive medication use), yet more than 75% of the sample population had NLRs (methylation-derived) lower than directly measured NLRs reported in a cohort with hypertension (39). It should be noted that, although validated and widely used (5), the methylation-derived cell deconvolution has been shown to underestimate neutrophil and overestimate lymphocyte proportions, by −1.66 and 0.4–1.0%, respectively (5). An average underestimation of the mdNLR vs. directly measured NLR of 0.6 units has also been reported (1). Consequently, such a 0.6-unit increase in mdNLR will shift our study population's median to just above the CVD risk cut-off (33), resulting in a reclassification of ~50% of the cohort being at increased risk, thereby aligning with the protein-based estimation (Supplementary Figure 1). Regardless of a possible adjustment, our data agrees with prior reports of more favorable NLRs in black populations (14), than Hispanic and white American ethnic groups. For the first time a comparison has been drawn between an African study population and European, Asian, and Chinese cohorts (13, 15, 16). No notable differences were observed between the black African study population reported on here and data from European, Asian, or Chinese cohorts.

In agreement with the adjusted mdNLR, the mdLMR also classified half of the PURE-SA-NW population as being at increased risk of CVD. Contrary to the mdNLR, no adjustment is required when comparing the mdLMR with directly measured ratios (5). On average, the PURE-SA-NW study population had lower mdLMRs than the LMRs previously reported for Asian, Chinese, and Western Indian cohorts (15, 16, 40). An overlap in the reference ranges of these four ethnic groups that were compared was, however, clearly visible (Supplementary Figure 2). These ranges, furthermore, spanned the LMRs reported in patients with coronary artery disease, coronary lesions, and chronic stable angina (34, 40, 41). It is noteworthy that, altogether, the studies that investigate LMR in the context of CVD represent fewer than N = 1 000 individuals of whom only N = 162 are controls (34). The limited evidence, together with the observation that the LMRs reported in four ostensibly healthy cohorts spanned, by a wide margin, the LMRs reported for CVD patients, highlights the need for more research on the LMR and its accuracy in risk prediction, because recent evidence suggested that the LMR might, in some instances, be more useful in CVD risk prediction than the NLR (34).

### Inflammation as a Contributor to Cardiovascular Risk

The central finding of this study is that, although current research relies heavily on protein-based inflammatory markers for CVD risk estimation (17, 25), methylation-derived inflammatory markers appear not only to be more strongly associated with CVF than protein-based markers but also independently so. Collectively, more variance was explained using a combination of methylation and protein markers, than the conventional protein markers on their own, suggesting that the methylation biomarkers could offer unique information on the inflammatory process and are not just surrogate markers for inflammatory proteins.

Prior evidence has shown a multitude of possible mechanisms through which individual leukocyte sub-types, proxied for in this study by methylation-derived cell count markers, may directly contribute to CVD (reviewed by 7, 34, 45). Neutrophils, for example, secrete inflammatory mediators and proteolytic enzymes related to vascular wall degeneration (7, 33). Macrophages (matured monocytes) contribute to cardiovascular risk mainly through its secretion of cytokines and reactive oxidative species once infiltrated to atherosclerotic plaque (34). On the other hand, regulatory T-cells (a lymphocyte sub-type) is a known role player in the development of hypertension through its role in the renin–angiotensin system (39).

The implication of these findings is that using a single protein marker (typically CRP, IL-6, or TNF-α in CVD-related investigations) to adjust for “inflammation” might not capture all the variance of the true inflammatory effect. The superiority of cell counts lies in their ability to reflect information about both the innate and complementary inflammatory processes. Adding to this advantage is the ability to provide methylation-derived cell count estimates retrospectively, from any well-preserved whole-blood or leukocyte samples where DNA is available, thus enabling the reinvestigation of large sample sets already collected.

### CpGs as an mdNLR Proxy

One previous study identified (4) and another replicated (2) the use of the five investigated CpGs as surrogates for the mdNLR in cancer case-control studies in American populations. Here, we evaluated their potential use in black South African men with low-grade inflammation associated with CVD risk. All five CpGs strongly associated with the mdNLR, confirming their robust associations with myeloid cell differentiation in a population-based study of a different ethnic group than previously reported. For arterial stiffness, cg25938803 was the most important contributor, even after the mdNLR and cg03621504 had been accounted for. Similarly, for SBP, cg03621504 contributed considerably (7.3%) to the explained variance, also after protein-based inflammatory markers and the mdNLR were added to the model. Apart from its strong associations with HR and arterial stiffness, cg03621504 was the only inflammatory biomarker with evidence of a possible association with BP, contributing 3–4% to the variance in all instances, at *p* < 0.05 (Supplementary Tables 3, 4). It is, therefore, possible that this CpG represents a novel marker of CVD risk.

Our observation that the myeloid CpGs contributed to CVF variance regardless of the prior inclusion of the mdNLR suggests that these CpGs may associate with CVF independently of cell-counts. This argument is strengthened by the fact that although the five CpGs were equally reflective of the mdNLR, associations with CVF markers differed in strength. Population-specific variance may contribute to the different patterns of association. Indeed, we found that two of the investigated CpGs, cg01591037 and cg00901982, are associated with *cis* single nucleotide polymorphisms (SNPs, rs76297553 and rs6546566, respectively) that are both methylation quantitative trait loci (mQTLs) for these CpGs and are expression QTLs for local genes (42, 43). Population differences in the minor allele frequencies of these SNPs (0.3% and 26% in African Americans compared to 6% and 30% in Europeans for rs76297553 and rs6546566, respectively) have been reported by the 1000 Genomes project (44).

### Strengths and Limitations

A limitation of this study is that we only investigated men, so we are unable to generalize our findings to women. Secondly, although methylation-derived cell ratios are accurate reflections of directly measured ratios, the lack of available literature on methylation-derived cut-offs for CVD risk and outcomes hindered direct comparison with directly measured ratios, particularly in the case of NLR. As a result of our limited sample size and the stringent statistical approach followed, there may be some true positive findings not emphasized here. For this reason, we reported all our results in the supplementary material so that our findings may be replicated in larger cohorts. Lastly, although we were able to provide evidence for the independent contribution of the myeloid CpGs to CVF variance and CVD risk, we were not able to further investigate these mechanisms.

The limitations mentioned above are met with various strengths. We were able to layer evidence from five inflammatory proteins frequently investigated in the context of CVD, with cytological data, whereas previous studies mostly had access to one or the other (3, 33, 45). This is particularly rare in an ostensibly healthy cohort. This is also the first time methylation-derived cell ratio estimates and the myeloid CpGs has been investigated in a Sub-Saharan African study population, which addresses the need for more ethnic representation and reference sets, particularly in epigenetic epidemiology (14, 15, 46). The lack of data on covariates known to affect cell count ratios, some even in a population-specific manner (14), such as adiposity, smoking, and medication use, has hindered previous investigations of population-specific reference ranges. We addressed this by investigating a richly phenotyped study population in whom we were able to identify and evaluate many, previously unstudied, potential confounding factors.

## Conclusion

The methylation-derived cell ratio estimates observed in this South African study population were comparable to previously investigated ostensibly healthy ethnic groups. The CVD risk reflected by these ratios was in accordance with that of CRP and several CVF and CVD risk markers. Five CpGs previously suggested as surrogates for the mdNLR in cancer patients were similarly highly associated with mdNLR in our cohort, regardless of the absence of overt inflammation. However, the contribution of these CpGs to CVF was independent from their effect on myeloid differentiation and robust to adjustment for known CVD risk factors, illustrating their potential functional relevance, apart from their role in myeloid differentiation. We demonstrate that population-specific genetic variance may contribute to these CpG-CVF associations even when comparable CpG-mdNLR relationships are observed. Methylation-derived and protein-based inflammatory biomarkers explain independent portions of CVF variance; the best characterization of CVF variance is obtained when methylation biomarkers, particularly the myeloid CpGs, are included in models containing known CVD risk markers, including protein-based inflammatory markers. Cell count data are highly valuable when the aim is to characterize inflammation, particularly when DNA is available, allowing the reinvestigation of existing cohort data and potentially circumventing the need for new data collection. The widely used Infinium HumanMethylation 450 K and EPIC BeadChip arrays include the myeloid CpGs reported here and methods to derive cell counts have been rigorously validated for these assays. Consequently, many large epigenetic epidemiology cohorts are already in possession of the necessary data to investigate cell count-related immunomodulation.

## Data Availability Statement

The datasets generated for this study will not be made publicly available. The data presented in this study are part of the larger South African and international PURE study and are therefore not under the sole jurisdiction of the corresponding author. Data will be made available upon reasonable request and with the permission of the Health Research Ethics Committee of the North-West University and the principal investigator of the PURE-SA-NW study, Prof. I.M. Kruger (lanthe.kruger@nwu.ac.za) at the North-West University's Africa Unit for Transdisciplinary Health Research.

## Ethics Statement

This study was reviewed and approved by the Health Research Ethics Committee of the Ethics Office for Research, Training and Support at the North-West University, South Africa. The participants provided their written informed consent to participate in this study.

## Author Contributions

HC, MP, HE, and CN-R conceptualized the study. Funding was acquired by MP and FG. AS acquired the cardiovascular function data. HC performed the data analysis and wrote the manuscript. HE and MP supervised the data analysis and interpreted the results with HC. All authors contributed to the critical review and editing of the manuscript.

## Conflict of Interest

The authors declare that the research was conducted in the absence of any commercial or financial relationships that could be construed as a potential conflict of interest.
