# Supplementary material for: Methylation vs. Protein Inflammatory Biomarkers and Their Associations With Cardiovascular Function
Source: Front Immunol. 2020 Jul 31;11:1577. doi: 10.3389/fimmu.2020.01577 (PMC7411149; doi:10.3389/fimmu.2020.01577)
Supplement: Supplementary file 1 [file Data_Sheet_1.docx]

Supplementary Material

**Supplementary Table 1** Descriptive characteristics of the study population

| **Clinical characteristics** | **Median**  **(25% ; 75%)** |
| --- | --- |
| **Blood cell type proportions** |  |
| B-cells (%) | 3.33 (2.51 ; 4.79) |
| CD4+ T-cells (%) | 10.7 (7.92 ; 14.3) |
| CD8+ T-cells (%) | 11.1 (7.18 ; 14.4) |
| Neutrophils (%) | 46.9 (39.7 ; 53.7) |
| Monocytes (%) | 8.94 (7.41 ; 10.8) |
| Natural killer cells (%) | 10.6 (8.90 ; 12.6) |
| **Education** [N(%)] |  |
| None | 26 (21.7) |
| 1–7 years of schooling | 66 (55.0) |
| 8–12 years of schooling | 28 (23.3) |
| **Alcohol consumption status** [N(%)] |  |
| Never user | 56 (46.7) |
| Ever user | 64 (53.3) |
| **Alcohol consumption*** (g/d) | 20.8 (5.61 ; 47.93) |
| **Duration of smoking** [N(%)^&^] |  |
| >10 years | 45 (70.3) |
| 5-10 years | 15 (23.4) |
| <5 years | 4 (6.3) |
| **CVD medication use** [N(%)] |  |
| Aspirin | 20 (16.7) |
| Statins | 4 (3.33) |
| **Anti-hypertensive medication incl.^#^** | 40 (33.3) |
| Diuretics | 34 (28.3) |
| Beta-blockers | 3 (2.5) |
| ACE inhibitors | 13 (10.8) |
| Calcium channel blockers | 13 (10.8) |
| **Gamma-glutamyl transferase** (U/L) | 40.6 (25.8 ; 78.4) |
| **Total cholesterol** (mmol/L) | 4.37 (3.64 ; 4.97) |
| **Triglycerides** (mmol/L) | 1.05 (0.80 ; 1.41) |
| **Markers of glucose metabolism** |  |
| Fasting glucose (mmol/L) | 5.04 (4.65 ; 5.55) |
| Glycated haemoglobin (mmol/mol) | 5.40 (5.10 ; 5.70) |
| **Waist circumference** (cm) | 81.6 (74.4 ; 93.5) |
| **Physical activity** (index) | 2.42 (1.94 ; 2.90) |

CVD: Cardiovascular disease. *Current users with available consumption data only, n = 50. ^&^ Limited to current users, n=64. ^#^Detailed summary of types of antihypertensive medication reported; participants may use more than one of these at a time.

**Supplementary Table 2** Partial Spearman correlations among protein-based and methylation-derived biomarkers of inflammation and markers of cardiovascular function

| **Inflammatory biomarker** | **SBP** | | **DBP** | | **PP** | **HR** | | | **cfPWV** |
| --- | --- | --- | --- | --- | --- | --- | --- | --- | --- |
|  | **Spearman Rho** | | | | | | | | |
| **IL-6** | –0.09 | –0.09 | | –0.09 | | | 0.14 | 0.09 | |
| **IL-10** | –0.08 | –0.01 | | –0.13 | | | 0.22 | 0.03 | |
| **TNF-α** | –0.05 | –0.04 | | –0.08 | | | 0.11 | 0.13 | |
| **IFN-γ** | 0.05 | –0.04 | | 0.08 | | | –0.06 | 0.18 | |
| **CRP** | –0.07 | –0.01 | | –0.09 | | | 0.23 | 0.03 | |
| **Score** | –0.06 | –0.04 | | –0.09 | | | 0.24 | 0.15 | |
| **mdNLR** | –0.07 | –0.04 | | –0.07 | | | 0.25 | 0.21 | |
| **mdLMR** | 0.11 | 0.07 | | 0.09 | | | –0.23 | –0.09 | |
| **cg25938803** | 0.11 | 0.04 | | 0.13 | | | **–0.27** | **–0.29** | |
| **cg10456459** | 0.07 | 0.02 | | 0.11 | | | **–0.30** | –0.15 | |
| **cg01591037** | 0.07 | 0.04 | | 0.07 | | | –0.13 | –0.16 | |
| **cg03621504** | 0.24 | 0.18 | | 0.22 | | | –0.25 | –0.18 | |
| **cg00901982** | 0.14 | 0.11 | | 0.12 | | | –0.22 | –0.18 | |

Data reported as Spearman’s rho values while controlling for age. Significant correlations are highlighted in bold using the Bonferroni threshold of p ≤ 0.004 (α = 0.05/12 tests, calculated as 4 independent inflammatory x 3 independent CVF markers). ‘Score’ represents the average of the IL-6, IL-10, TNA-α, IFN-γ and CRP z-scores. CRP: C-reactive protein; IFN-γ: interferon-gamma; IL-6: interleukin-6; IL-10: interleukin-10; mdLMR: methylation-derived lymphocyte-to-monocyte ratio; mdNLR: methylation-derived neutrophil-to-lymphocyte ratio; TNF-α: tumor necrosis factor-alpha.

**Supplementary Table 3** Variance in cardiovascular function explained by individual inflammatory biomarkers

| **Inflammatory biomarker** | **Model** | **CVF variance explained by covariates** | **Inflammatory biomarker’s contribution to the model** | | **Variance explained by full model** |
| --- | --- | --- | --- | --- | --- |
|  |  |  | **β (25% ; 75%)** | **p** |  |
| **SBP** (log mmHg) | | | | | |
| **IL-6** | 1 | 5% | –0.01 (–0.04 ; 0.02) | 0.49 | 7% |
|  | 2 | 12% | –0.01 (–0.04 ; 0.02) | 0.60 | 13% |
| **IL-10** | 1 | 5% | 0.02 (–0.06 ; 0.09) | 0.68 | 5% |
|  | 2 | 12% | 0.01 (–0.07 ; 0.09) | 0.86 | 12% |
| **TNF-α** | 1 | 5% | 0.05 (–0.03 ; 0.13) | 0.21 | 7% |
|  | 2 | 12% | 0.05 (–0.04 ; 0.13) | 0.27 | 13% |
| **IFN-γ** | 1 | 5% | 0.02 (–0.01 ; 0.05) | 0.27 | 7% |
|  | 2 | 12% | 0.02 (–0.02 ; 0.05) | 0.30 | 13% |
| **CRP** | 1 | 5% | –0.01 (–0.03 ; 0.02) | 0.59 | 7% |
|  | 2 | 12% | –0.01 (–0.03 ; 0.02) | 0.66 | 13% |
| **Score** | 1 | 5% | –1.70 (–7.39 ; 4.34) | 0.71 | 7% |
|  | 2 | 12% | –1.07 (–6.53 ; 4.70) | 0.57 | 14% |
| **mdNLR** | 1 | 5% | –0.03 (–0.1 ; 0.03) | 0.34 | 6% |
|  | 2 | 12% | –0.04 (–0.1 ; 0.03) | 0.26 | 13% |
| **mdLMR** | 1 | 5% | 0.08 (–0.03 ; 0.19) | 0.14 | 7% |
|  | 2 | 12% | 0.08 (–0.03 ; 0.19) | 0.17 | 13% |
| **cg25938803** | 1 | 5% | 0.07 (–0.05 ; 0.2) | 0.26 | 6% |
|  | 2 | 12% | 0.09 (–0.04 ; 0.2) | 0.17 | 13% |
| **cg10456459** | 1 | 5% | 0.08 (–0.03 ; 0.18) | 0.16 | 7% |
|  | 2 | 12% | 0.08 (–0.03 ; 0.18) | 0.13 | 14% |
| **cg01591037** | 1 | 5% | 14.3 (–16.7 ; 56.9) | 0.41 | 6% |
|  | 2 | 12% | 17.7 (–14.8 ; 62.6) | 0.32 | 12% |
| **cg03621504** | 1 | 5% | 0.10 (0.02 ; 0.17) | 0.01 | 11% |
|  | 2 | 12% | 0.10 (0.02 ; 0.17) | 0.01 | 17% |
| **cg00901982** | 1 | 5% | 23.7 (–8.77 ; 67.7) | 0.17 | 7% |
|  | 2 | 12% | 26.0 (–7.77 ; 72.0) | 0.14 | 13% |
| **DBP** (mmHg) | | | | | |
| **IL-6** | 1 | 0% | –0.01 (–0.03 ; 0.01) | 0.38 | 1% |
|  | 2 | 12% | –0.01 (–0.03 ; 0.01) | 0.44 | 13% |
| **IL-10** | 1 | 0% | 0.01 (–0.04 ; 0.06) | 0.73 | 1% |
|  | 2 | 12% | 0.001 (–0.05 ; 0.06) | 0.97 | 12% |
| **TNF-α** | 1 | 0% | 0.02 (–0.04 ; 0.08) | 0.52 | 1% |
|  | 2 | 12% | 0.01 (–0.05 ; 0.07) | 0.75 | 12% |
| **IFN-γ** | 1 | 0% | –0.002 (–0.03 ; 0.02) | 0.86 | 1% |
|  | 2 | 12% | –0.01 (–0.03 ; 0.02) | 0.61 | 12% |
| **CRP** | 1 | 0% | 0.004 (–0.02 ; 0.02) | 0.68 | 1% |
|  | 2 | 12% | 0.004 (–0.02 ; 0.02) | 0.71 | 12% |
| **Score** | 1 | 0% | –0.63 (–4.77 ; 3.52) | 0.77 | 1% |
|  | 2 | 12% | –1.68 (–5.89 ; 3.52) | 0.43 | 13% |
| **mdNLR** | 1 | 0% | –0.01 (–0.06 ; 0.03) | 0.55 | 1% |
|  | 2 | 12% | –0.02 (–0.07 ; 0.02) | 0.34 | 12% |
| **mdLMR** | 1 | 0% | 0.03 (–0.04 ; 0.11) | 0.39 | 1% |
|  | 2 | 12% | 0.03 (–0.05 ; 0.10) | 0.47 | 12% |
| **cg25938803** | 1 | 0% | 0.02 (–0.07 ; 0.11) | 0.62 | 1% |
|  | 2 | 12% | 0.05 (–0.04 ; 0.14) | 0.25 | 13% |
| **cg10456459** | 1 | 0% | 0.04 (–0.04 ; 0.11) | 0.35 | 1% |
|  | 2 | 12% | 0.05 (–0.03 ; 0.12) | 0.20 | 13% |
| **cg01591037** | 1 | 0% | 9.62 (–12.8 ; 32.0) | 0.40 | 1% |
|  | 2 | 12% | 13.6 (–8.54 ; 32.0) | 0.23 | 13% |
| **cg03621504** | 1 | 0% | 0.06 (0.01 ; 0.11) | 0.03 | 4% |
|  | 2 | 12% | 0.06 (0.01 ; 0.11) | 0.03 | 15% |
| **cg00901982** | 1 | 0% | 15.3 (–6.20 ; 36.8) | 0.16 | 2% |
|  | 2 | 12% | 17.1 (–4.23 ; 36.8) | 0.12 | 14% |
| **PP** (log mmHg) | | | | | |
| **IL-6** | 1 | 9% | –0.01 (–0.07 ; 0.04) | 0.60 | 12% |
|  | 2 | 12% | –0.01 (–0.06 ; 0.04) | 0.78 | 14% |
| **IL-10** | 1 | 9% | 0.01 (–0.12 ; 0.14) | 0.87 | 8% |
|  | 2 | 12% | 0.001 (–0.14 ; 0.14) | 0.98 | 11% |
| **TNF-α** | 1 | 9% | 0.08 (–0.06 ; 0.22) | 0.28 | 10% |
|  | 2 | 12% | 0.08 (–0.07 ; 0.22) | 0.28 | 13% |
| **IFN-γ** | 1 | 9% | 0.06 (0.00 ; 0.11) | 0.06 | 12% |
|  | 2 | 12% | 0.06 (0.00 ; 0.11) | 0.04 | 16% |
| **CRP** | 1 | 9% | –0.03 (–0.07 ; 0.02) | 0.25 | 12% |
|  | 2 | 12% | –0.02 (–0.07 ; 0.02) | 0.34 | 15% |
| **Score** | 1 | 9% | –1.98 (–10.89 ; 7.82) | 0.68 | 12% |
|  | 2 | 12% | –1.54 (–11.1 ; 9.06) | 0.76 | 14% |
| **mdNLR** | 1 | 9% | –0.06 (–0.17 ; 0.05) | 0.29 | 10% |
|  | 2 | 12% | –0.06 (–0.17 ; 0.05) | 0.32 | 13% |
| **mdLMR** | 1 | 9% | 0.14 (–0.04 ; 0.33) | 0.13 | 10% |
|  | 2 | 12% | 0.14 (–0.06 ; 0.33) | 0.16 | 14% |
| **cg25938803** | 1 | 9% | 0.15 (–0.07 ; 0.36) | 0.17 | 10% |
|  | 2 | 12% | 0.14 (–0.09 ; 0.36) | 0.23 | 13% |
| **cg10456459** | 1 | 9% | 0.14 (–0.05 ; 0.32) | 0.15 | 10% |
|  | 2 | 12% | 0.13 (–0.06 ; 0.32) | 0.18 | 13% |
| **cg01591037** | 1 | 9% | 23.2 (–29.8 ; 116) | 0.42 | 9% |
|  | 2 | 12% | 24.8 (–27.4 ; 115) | 0.46 | 12% |
| **cg03621504** | 1 | 9% | 0.14 (0.02 ; 0.27) | 0.03 | 12% |
|  | 2 | 12% | 0.15 (0.01 ; 0.27) | 0.03 | 16% |
| **cg00901982** | 1 | 9% | 26.6 (–25.0 ; 114) | 0.37 | 9% |
|  | 2 | 12% | 29.1 (–25.0 ; 122) | 0.35 | 13% |
| **HR** (log bpm) | | | | | |
| **IL-6** | 1 | 0% | 0.02 (–0.02 ; 0.05) | 0.34 | 2% |
|  | 2 | 9% | 0.02 (–0.02 ; 0.05) | 0.30 | 12% |
| **IL-10** | 1 | 0% | 0.09 (0.00 ; 0.17) | 0.04 | 4% |
|  | 2 | 9% | 0.11 (0.02 ; 0.17) | 0.02 | 14% |
|  |  |  |  |  |  |
| **TNF-α** | 1 | 0% | 0.04 (–0.06 ; 0.13) | 0.44 | 1% |
|  | 2 | 9% | 0.05 (–0.05 ; 0.13) | 0.34 | 11% |
| **IFN-γ** | 1 | 0% | –0.02 (–0.06 ; 0.02) | 0.25 | 1% |
|  | 2 | 9% | –0.03 (–0.07 ; 0.02) | 0.17 | 12% |
| **CRP** | **1** | **0%** | **0.04 (0.01 ; 0.07)** | **0.006** | **7%** |
|  | **2** | **9%** | **0.05 (0.02 ; 0.07)** | **0.001** | **19%** |
| **Score** | 1 | 0% | 5.96 (–0.79 ; 13.2) | 0.08 | 3% |
|  | 2 | 9% | 7.20 (0.05 ; 14.9) | 0.05 | 13% |
| **mdNLR** | **1** | **0%** | **0.11 (0.04 ; 0.18)** | **0.003** | **7%** |
|  | **2** | **9%** | **0.10 (0.02 ; 0.18)** | **0.01** | **15%** |
| **mdLMR** | **1** | **0%** | **–0.19 (–0.32 ; –0.07)** | **0.003** | **8%** |
|  | **2** | **9%** | **–0.18 (–0.30 ; –0.07)** | **0.006** | **16%** |
| **cg25938803** | 1 | 0% | –0.20 (–0.35 ; –0.06) | 0.005 | 7% |
|  | 2 | 9% | –0.15 (–0.30 ; –0.06) | 0.05 | 13% |
| **cg10456459** | **1** | **0%** | **–0.20 (–0.32 ; –0.08)** | **0.002** | **8%** |
|  | **2** | **9%** | **–0.17 (–0.29 ; –0.08)** | **0.01** | **15%** |
| **cg01591037** | 1 | 0% | ­–25.8 (–48.6 ; 7.24) | 0.11 | 2% |
|  | 2 | 9% | –18.7 (–44.2 ; 18.6) | 0.28 | 10% |
| **cg03621504** | **1** | **0%** | **–0.12 (–0.21 ; –0.04)** | **0.004** | **7%** |
|  | **2** | **9%** | **–0.11 (–0.20 ; –0.04)** | **0.02** | **14%** |
| **cg00901982** | 1 | 0% | –35.2 (–54.3 ; –7.95) | 0.02 | 5% |
|  | 2 | 9% | –31.2 (–52.0 ; –1.38) | 0.04 | 13% |
| **cfPWV** (log bpm) | | | | | |
| **IL-6** | 1 | 25% | 0.02 (–0.01 ; 0.05) | 0.16 | 26% |
|  | 2 | 36% | 0.02 (–0.01 ; 0.05) | 0.19 | 38% |
| **IL-10** | 1 | 25% | 0.03 (–0.04 ; 0.09) | 0.48 | 26% |
|  | 2 | 36% | 0.04 (–0.02 ; 0.09) | 0.20 | 38% |
| **TNF-α** | 1 | 25% | 0.04 (–0.03 ; 0.12) | 0.28 | 27% |
|  | 2 | 36% | 0.06 (–0.02 ; 0.12) | 0.13 | 40% |
| **IFN-γ** | 1 | 25% | 0.03 (–0.01 ; 0.06) | 0.12 | 27% |
|  | 2 | 36% | 0.02 (–0.01 ; 0.06) | 0.23 | 40% |
| **CRP** | 1 | 25% | 0.01 (–0.01 ; 0.03) | 0.41 | 25% |
|  | 2 | 36% | 0.02 (–0.01 ; 0.03) | 0.13 | 38% |
| **Score** | 1 | 25% | 4.33 (–1.26 ; 10.2) | 0.13 | 26% |
|  | 2 | 36% | 5.97 (0.41 ; 11.85) | 0.04 | 41% |
| **mdNLR** | 1 | 25% | 0.07 (0.01 ; 0.14) | 0.02 | 29% |
|  | 2 | 36% | 0.06 (0.00 ; 0.14) | 0.05 | 39% |
| **mdLMR** | 1 | 25% | –0.12 (–0.22 ; –0.02) | 0.02 | 28% |
|  | 2 | 36% | –0.08 (–0.18 ; –0.02) | 0.11 | 38% |
| **cg25938803** | **1** | **25%** | **–0.20 (–0.31 ; –0.09)** | **3.8E–04** | **33%** |
|  | **2** | **36%** | **–0.18 (–0.29 ; –0.09)** | **0.002** | **42%** |
| **cg10456459** | 1 | 25% | –0.10 (–0.21 ; 0.00) | 0.05 | 27% |
|  | 2 | 36% | –0.08 (–0.18 ; 0.00) | 0.12 | 38% |
| **cg01591037** | 1 | 25% | –24.5 (–44.4 ; 2.40) | 0.07 | 27% |
|  | 2 | 36% | –16.9 (–38.5 ; 12.2) | 0.22 | 37% |
| **cg03621504** | **1** | **25%** | **–0.12 (–0.19 ; –0.04)** | **0.002** | **31%** |
|  | **2** | **36%** | **–0.09 (–0.16 ; –0.04)** | **0.01** | **40%** |
| **cg00901982** | 1 | 25% | –32.1 (–49.0 ; –9.58) | 0.009 | 30% |
|  | 2 | 36% | –25.0 (–43.4 ; –0.55) | 0.05 | 39% |

*With the exception of cg00901982, cg01591037 and DBP all inflammatory and cardiovascular biomarkers were log_e_-transformed prior to analysis. Linear-log and log-linear regression coefficients are presented as marginal effects. For log(CVF marker/‘y’) ~ log(inflammatory marker/‘x’), interpretation should be that one per cent change in x will induce a regression coefficient (β) per cent change in y. For log(y)~x, interpretation should be that one unit change in x will induce a β per cent change in y. For y~log(x), interpretation should be one per cent change in x results in a β unit change in y. ‘Score’ represents the average of the IL-6, IL-10, TNA-α, IFN-γ and CRP z-scores (derived from log_e_-transformed data for all). **Model 1:** CVF marker ~ (inflammatory biomarker) + age; **Model 2:** CVF marker ~ (inflammatory biomarker) + age + smoking status + dwelling place + smoking status + BMI + LDL-C + HDL-C + medication use. When cfPWV was the outcome, mean arterial pressure was additionally adjusted for. p ≤ 0.004 highlighted in bold. DBP: diastolic blood pressure; SBP: systolic blood pressure; PP: pulse pressure; CRP: C-reactive protein; cfPWV: carotid-femoral pulse wave velocity; IFN-γ: interferon-gamma; IL-6: interleukin-6; IL-10: interleukin-10; IQR: interquartile range; mdLMR: methylation-derived lymphocyte-to-monocyte ratio; mdNLR: methylation-derived neutrophil-to-lymphocyte ratio; HR: heart rate; TNF-α: tumour necrosis factor-alpha.

**Supplementary Table 4** The additive value of methylation-derived inflammatory biomarkers to known cardiovascular risk markers in relation to cardiovascular function

| **Regression model*** | **Inflammatory biomarker** | | | **Total variance explained** | ***Χ^2^***  ***p-value*** |
| --- | --- | --- | --- | --- | --- |
|  | ***β* (25% ; 75%)** | **p** | **Contribution to CVF variance**^&^ |  |  |
| **SBP** (log mmHg) | | | | | |
| Model 3 |  |  |  | 14% | **0.005** |
| +mdNLR | 0.11 (0 ; 0.23) | 0.05 | 2.2% | **22%** |  |
| +cg03621504 | 0.21 (0.08 ; 0.35) | **0.003** | 7.3% |  |  |
| **DBP** (mmHg) | | | | | |
| Model 3 |  |  |  | 13% | 0.03 |
| +mdNLR | 0.06 (–0.02 ; 0.01) | 0.15 | 1.5% | 19% |  |
| + cg03621504 | 0.01 (0.02 ; 0.02) | **0.02** | 5.4% |  |  |
| **PP** (log mmHg) | | | | | |
| Model 3 |  |  |  | 14% | 0.03 |
| +mdNLR | 0.17 (–0.03; 0.37) | 0.10 | 1.4% | 20% |  |
| + cg03621504 | 0.30 (0.06 ; 0.54) | **0.01** | 4.5% |  |  |
| **HR** (log bpm) | | | | | |
| Model 3 |  |  |  | 13% | 0.04 |
| + cg25938803 | –0.09 (–0.31 ; 0.14) | 0.44 | 2.0% | 20% |  |
| + cg10456459 | –0.27 (–0.50 ; –0.05) | **0.02** | 4.8% |  |  |
| + cg01591037 | 111.7 (7.25 ; 322) | 0.03 | 2.2% |  |  |
| **cfPWV** (log m/s) | | | | | |
| Model 3 |  |  |  | 41% | **0.008** |
| +mdNLR | –0.13 (–0.26 ; –0.003) | 0.05 | 1.5% | **48%** |  |
| + cg25938803 | –0.24 (–0.43 ; –0.06) | **0.01** | 4.7% |  |  |
| + cg03621504 | –0.11 (–0.24 ; 0.02) | 0.10 | 1.6% |  |  |

*With the exception of cg00901982, cg01591037 and DBP all inflammatory and cardiovascular biomarkers were loge-transformed prior to analysis. Linear-log and log-linear regression coefficients are presented as marginal effects. For log(CVF marker/‘y’) ~ log(inflammatory marker/‘x’), interpretation should be that one per cent change in x will induce a regression coefficient (β) per cent change in y. For log(y)~x, interpretation should be that one unit change in x will induce a β per cent change in y. For y~log(x), interpretation should be one per cent change in x results in a β unit change in y. The ^&^*lmg* metric providing a decomposition of the model explained variance into non-negative contributions (Grömping, 2006). Χ^2^ p value = Chi-square p value when the regression models with and without methylation-derived inflammatory biomarkers are compared. ***Model 3:** CVF marker ~ age + smoking status + dwelling area + BMI + LDL-C + HDL-C + medicine use + score (the average of the IL-6. IL-10. TNA-α. IFN-γ and CRP z-scores). When cfPWV was the outcome. mean arterial pressure was additionally adjusted for. p ≤ 0.02 highlighted in bold. DBP: diastolic blood pressure; SBP: systolic blood pressure; PP: pulse pressure; cfPWV: carotid-femoral pulse wave velocity; mdNLR: methylation-derived neutrophil-to-lymphocyte ratio.


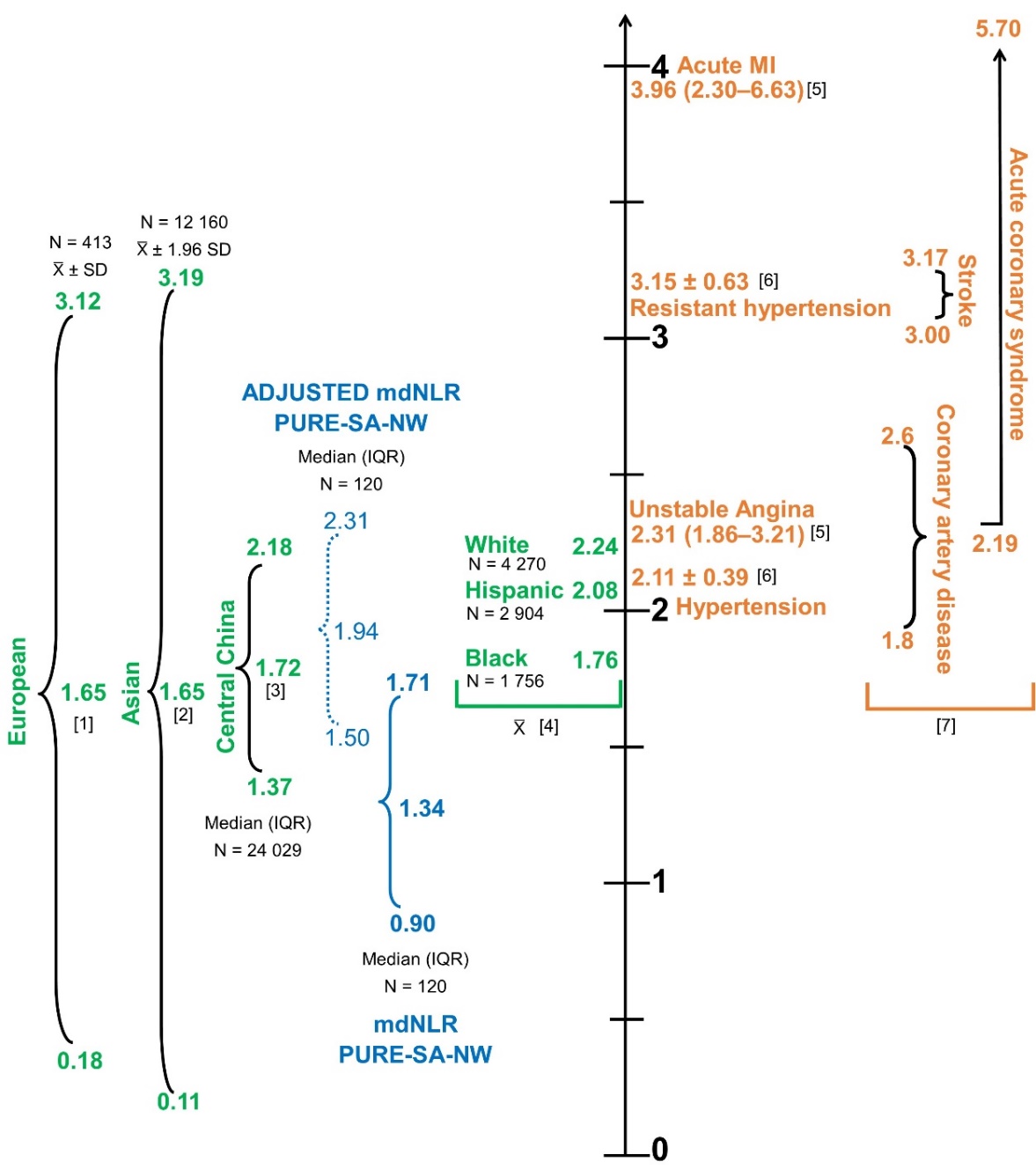


**Supplementary Figure 1** PURE-SA-NW methylation-derived NLR compared to previously published directly measured NLR reference ranges for healthy and at-risk groups

IQR: interquartile range; md: methylation derived; NLR: neutrophil-to-lymphocyte ratio; PURE-SA-NW: Prospective urban and rural epidemiology study cohort in South Africa’s North West province. Adjusted mdNLR: mdNLR + 0.6 – accounting for reported differences in methylation-derived and directly measured NLR (Koestler et al. (2016), see Discussion). Only evidence relating to cardiovascular disease risk is reported. Higher NLRs are associated with poor survival. References: [1] Forget et al. (2017); [2] Lee et al. (2018), [3] Meng et al. (2018); [4] Azab et al. (2014); [5] Tahto et al. (2017); [6] Belen et al. (2015); [7] Angkananard et al. (2018). Reference 4 reports on cohorts from the United States. Black and White refer to self-identified non-hispanic black and non-hispanic white groups. References 5 and 6 report mean ± SD of groups (n = 50) defined by specific diseases. Reference 7 is a meta-analysis reporting on the cut-offs used when evaluating the odds of CVD outcomes; multiple studies of different group sizes are reported.


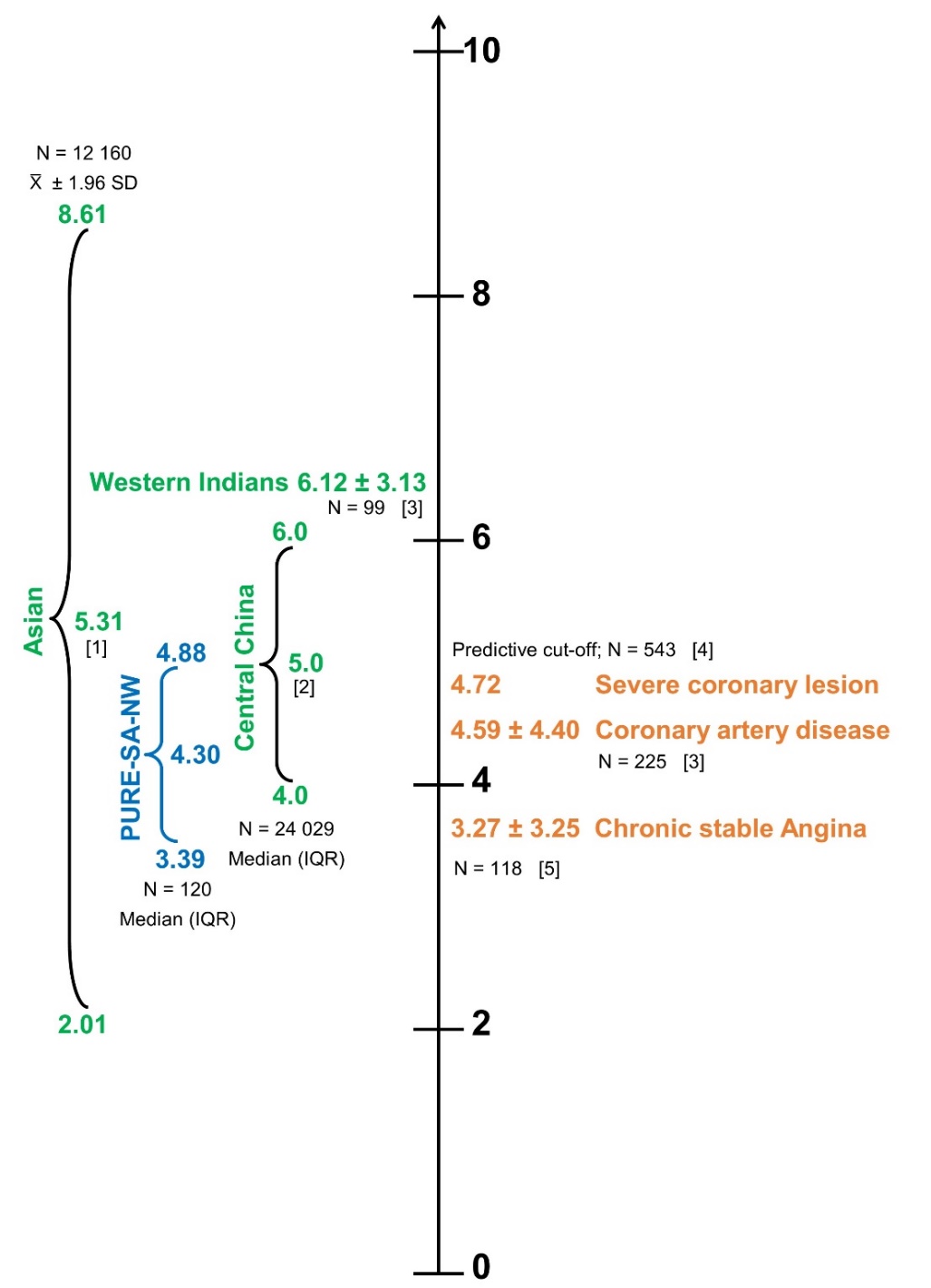


**Supplementary Figure 2** PURE-SA-NW methylation-derived LMR compared to previously published directly measured LMR ranges for healthy and at-risk groups

IQR: interquartile range; LMR: lymphocyte-to-monocyte ratio; Prospective urban and rural epidemiology study cohort in South Africa’s North West province. Only evidence relating to cardiovascular disease risk is reported. Lower LMRs are associated with poor survival. References: [1] Lee et al. (2018); [2] Meng et al. (2018); [3] Sharma et al. (2017); [4] Ji et al. (2017); [5] Zouridakis et al. (2000).

**References**

ANGKANANARD, T., ANOTHAISINTAWEE, T., MCEVOY, M., ATTIA, J. & THAKKINSTIAN, A. 2018. Neutrophil lymphocyte ratio and cardiovascular disease risk: a systematic review and meta-analysis. *BioMed research international*, 2018.

AZAB, B., CAMACHO-RIVERA, M. & TAIOLI, E. 2014. Average values and racial differences of neutrophil lymphocyte ratio among a nationally representative sample of United States subjects. *PloS one,* 9**,** e112361.

BELEN, E., SUNGUR, A., SUNGUR, M. A. & ERDOĞAN, G. 2015. Increased neutrophil to lymphocyte ratio in patients with resistant hypertension. *The journal of clinical hypertension,* 17**,** 532-537.

FORGET, P., KHALIFA, C., DEFOUR, J.-P., LATINNE, D., VAN PEL, M.-C. & DE KOCK, M. 2017. What is the normal value of the neutrophil-to-lymphocyte ratio? *BMC research notes,* 10**,** 12.

GRÖMPING, U. 2006. Relative importance for linear regression in R: the package relaimpo. *Journal of statistical software,* 17**,** 1-27.

JI, H., LI, Y., FAN, Z., ZUO, B., JIAN, X., LI, L. & LIU, T. 2017. Monocyte/lymphocyte ratio predicts the severity of coronary artery disease: a syntax score assessment. *BMC Cardiovascular disorders,* 17**,** 90.

KOESTLER, D. C., USSET, J., CHRISTENSEN, B. C., MARSIT, C. J., KARAGAS, M. R., KELSEY, K. T. & WIENCKE, J. K. 2016. DNA methylation-derived neutrophil-to-lymphocyte ratio: an epigenetic tool to explore cancer inflammation and outcomes. *Cancer epidemiology and prevention biomarkers*.

LEE, J. S., KIM, N. Y., NA, S. H., YOUN, Y. H. & SHIN, C. S. 2018. Reference values of neutrophil-lymphocyte ratio, lymphocyte-monocyte ratio, platelet-lymphocyte ratio, and mean platelet volume in healthy adults in South Korea. *Medicine,* 97**,** e11138-e11138.

MENG, X., CHANG, Q., LIU, Y., CHEN, L., WEI, G., YANG, J., ZHENG, P., HE, F., WANG, W. & MING, L. 2018. Determinant roles of gender and age on SII, PLR, NLR, LMR and MLR and their reference intervals defining in Henan, China: A posteriori and big‐data‐based. *Journal of clinical laboratory analysis,* 32**,** e22228.

SHARMA, K., PATEL, A. K., SHAH, K. H. & KONAT, A. 2017. Is neutrophil-to-lymphocyte ratio a predictor of coronary artery disease in Western Indians? *International journal of inflammation,* 2017.

TAHTO, E., JADRIC, R., POJSKIC, L. & KICIC, E. 2017. Neutrophil-to-lymphocyte ratio and its relation with markers of inflammation and myocardial necrosis in patients with acute coronary syndrome. *Medical archives,* 71**,** 312.

ZOURIDAKIS, E. G., GARCIA-MOLL, X. & KASKI, J. C. 2000. Usefulness of the blood lymphocyte count in predicting recurrent instability and death in patients with unstable angina pectoris. *The American journal of cardiology,* 86**,** 449.
